# Serum Leptin Level Is Reduced in Non-Obese Subjects with Type 2 Diabetes

**DOI:** 10.5812/ijem.6535

**Published:** 2012-12-21

**Authors:** Ghorban Mohammadzadeh, Nosratollah Zarghami

**Affiliations:** 1 Hyperlipidemia Research Center, Department of Clinical Biochemistry, Faculty of Medicine, Ahvaz Jundishapur University of Medical Sciences, Ahvaz, IR Iran; 2 Drug Applied Research Center, Tabriz University of Medical Sciences, Tabriz, IR Iran

**Keywords:** Leptin, Type 2 Diabetes, Body Mass Index

## Abstract

**Background:**

Leptin, a protein released from adipose tissue, could have significant role in pathogenesis of obesity and type 2 diabetes mellitus.

**Objectives:**

This study aimed to evaluate variations in serum leptin levels in non-obese subjects with type 2 diabetes mellitus (T2DM).

**Patients and Methods:**

We studied forty-one patients with type 2 diabetes. Fasting lipid profile, Hemoglobin A1c (HbA1c), serum leptin, insulin, and glucose levels were measured by standard methods.

**Results:**

The serum leptin level in type 2 diabetic patients (19.32 ± 11.43 ng/mL) was significantly lower than that in non-diabetic subjects (32.16 ± 11.02 ng/mL). Serum leptin level was strongly and positively correlated with body mass index (BMI) (r = 0.658, P < 0.0001) and calculated body fat percentage (r = 0.431, P < 0.0001) in all the study subjects with a better corrlation in the control subjcts compared to control cases (r = 0.661 for BMI and r = 0.466 for body fat). On the other hand, leptin showed a positive and significant correlation with insulin and HOMA- β (homeostasis model assessment for β-cell function) in both groups. Furthermore, leptin related to homeostasis model assessment for insulin resistance (HOMA-IR) (r = 0.422, P = 0.006) was observed only in T2DM subjects. Leptin showed negative correlation with waist to hip ratio in diabetic (r = -0.407, P =0.008) and non-diabetic subjects (r = -0.318, P =0.049). In the regression model, BMI, HOMA-β, and gender were independent predictors of leptin in all subjects. However, in non-diabetic and diabetic subjects, β-cell function and insulin were independent predictors, respectively (P =0.01).

**Conclusions:**

It is speculated that lower serum leptin levels in diabetic patients may be a consequence of male gender. Moreover, results suggest that serum leptin level in women is influenced differently than that in men.

## 1. Background

Diabetes mellitus comprises a group of metabolic disorders characterized by chronic hyperglycemia. Type 2 diabetes and its complications impose a tremendous burden both on individuals with diabetes and on healthcare systems. Leptin, the product of ob gene, is a peptide that is strongly correlated with adiposity and is a potential determinant of obesity and its complications. Leptin together with other adipocytokines affect insulin sensitivity and is accepted to play a role in pathogenesis of obesity-related disorders (1). Increased level of serum leptin is considered as a component of metabolic syndrome (2). It was suggested that resistance to leptin in β-cells might prevent the inhibitory effect of leptin on insulin secretion resulting in hyperinsulinemia, which might exhaust pancreatic β-cells leading to development of T2DM (3). Leptin is associated with body mass index (BMI) and body fat in non-obese and obese subjects and in patients with Type 2 diabetes mellitus (4). Serum leptin concentration also has a gender dimorphism, with higher serum levels in women than that in men (5, 6). Although, leptin levels are increased in obesity (5), obese subjects with type 2 diabetes display reduced leptin levels (7-9) which may be due to altered fat distribution (10). On the other hand, data collected from several previous studies have reported increased (11) or unchanged (12) leptin levels in diabetic patients. However, data regarding variations in leptin levels in non-obese subjects with T2DM are controversial, and to our knowledge, the association between leptin levels and anthropometric and clinical characteristics of T2DM in non-obese subjects has not been previously reported in Iranian subjects.

## 2. Objectives

In the current study, we aimed to report the variation of serum leptin levels in T2DM and the association between these levels and anthropometric and clinical characteristics of T2DM in comparison with which were seen in a healthy control group of non-diabetic subjects.

## 3. Patients and Methods

### 3.1. Study Patients

Forty-one middle-aged non-obese Iranian individuals with type 2 diabetes (21 women and 20 men, aged 42.09 ± 6.07 years) who consecutively visited the out-patient clinics for diabetes mellitus in hospitals of Sina University ,Tabriz, Iran, from October 2008 to January 2009 were enrolled in the present study. Type 2 diabetes was defined based on history of patients taking oral hypoglycemic drugs or according to the classification of American Diabetes Association as showing fasting plasma glucose concentration more than 126 mg/dL (13). Diabetic patients were treated by oral hypoglycemic agents (metformin, n=30; glibenclamide, n=11). No patients received insulin therapy. None of the subjects suffered from significant renal, hepatic, or cardiovascular diseases. The duration of diabetes was 1 to 6 years (mean: 2.50 ± 1.44 years). Patients did not consume alcohol or perform heavy exercises for at least one week before the study. 

The non-diabetic control group consisted of 39 middle-aged non-obese individuals (21 women and 18 men, aged 40.07 ± 7.29 years) who had received an annual health check-up. To select the non-diabetic control individuals, the following criteria were used: 1) No diabetes in their first degree relatives. 2) Fasting plasma glucose concentration less than 110 mg/dL. 3) Hemoglobin A1c concentration less than 5.5%. Non-diabetic subjects with endocrine disease, significant renal or hepatic diseases, and those receiving medications that control glucose metabolism, hypertension or hyperlipidemia were excluded from the study. Non-obesity was defined according to WHO criteria (BMI < 30 kg/m^2^).

### 3.2. Anthropometric Evaluation

Anthropometric indices including height, weight, and hip and waist circumferences were taken while subjects were in the standing position and wearing light clothing without shoes. Body weight and height were measured in kilograms and in centimeters, respectively. The waist circumference was taken at the smallest standing horizontal circumference between lower edge of rib cage and iliac crest; the hip circumference was taken at the largest standing horizontal circumference of the buttocks. Waist to hip ratio (WHR) was also calculated from the ratio of waist circumference in centemetrs to hip circumference in centemetrs as waist circumference divided by hip circumference. These parameters were measured by well-trained dietitians. The subjects were underwent a detailed examination by the medical office to assess the health status. The study was reviewed and approved by the institutional Ethics Committee of Tabriz University of Medical Sciences and written informed consent was obtained from all subjects after the explanation of the procedure.

### 3.3. Biochemical Analysis

AVenous blood samples (5mL from each) were drawn from all subjects who referred after 12-hour overnight fasting. Plasma glucose concentration was measured by the glucose oxidase method. Total cholesterol, triglycerides, and high-density lipoprotein-Cholesterol (HDL-C) were also measured. The low-density lipoprotein (LDL-C) cholesterol level was calculated using the Friedewald formula (LDL cholesterol = total cholesterol - HDL cholesterol - 1/5 triglycerides) in subjects with serum triglyceride concentrations less than 400 mg/ mL. Fasting serum insulin was measured by enzyme immunoassay using human insulin ELISA kit (Q-1-DiaPlus, USA) after the serum samples were thawed at room temperature. This assay had a sensitivity margin of 0. 5 µIU/mL. Intra- and inter-assay coefficients of variation were 6.45 and 6.45%, respectively. Hemoglobin A1c was measured in whole blood samples immediately after the collection according to boronate affinity assay by NycoCard (Axis-sheild , Norway) HbA1c protocol with the coefficient of variation (CV) below 5%. According to the percentage of HbA1c, diabetic group was divided into a well- controlled (patients with HbA1c below 7%, n= 25) and a poorly-controlled (patients with HbA1c above 7%, n= 16) subgroup. Serum leptin concentration was measured by enzyme-linked immunosorbent assay (ELISA) with a commercially available human leptin ELISA kit (Bio Vendor Laboratory Medicine, Inc., GmbH) using specific human leptin antibody. The intra- and inter-assay coefficients of variation were less than 5% for leptin. Before the assay, quality controls and all sera were diluted ⅓ times with a diluting buffer.

### 3.4. Calculations 

Body mass index (BMI) was calculated as weight (kilograms) divided by height squared (in square meters). Body fat content (BF %) was calculated according to the method of Lean et al. (14) which was shown to correlate with the percentage of body fat measured via underwater weighting (11) using the following formula:

Body fat% (men) = [(0.567 × waist circumference in cm) + (0.101 × age in years)] − 31.8

Body fat% (women) = [(0.438 × waist circumference in cm) + (0.221 × age in years)] − 9.4

Serum leptin levels have been correlated with body fat % calculated by above formulas (15). 

It was important to measure insulin resistance because it plays a role in the development of metabolic syndrome and diabetes mellitus. Although, in clinical practice many investigators consider hyperinsulinemic euglycemic clamp (16) and steady-state plasma glucose (17) value as ''gold standards'' to estimate insulin resistance, they are very complicated methods because of requirement to simultaneous infusions of insulin and glucose and multiple blood sampling for a period of 3 hours. A simple index of insulin sensitivity based on fasting glucose and insulin concentrations, such as homeostatic model assessment for insulin resistance (HOMA-IR), is easily obtained and may be a useful tool for large epidemiologic studies (18). 

HOMA calculation is based on the assumption that the degree of basal hyperglycemia is determined by a combination of β-cell deficiency and insulin resistance. Thus, we calculated pancreatic β-cell function and insulin resistance (IR) by glucose and insulin concentrations using homeostatic model assessment (HOMAβ-cell function and HOMA-IR, respectively) (19) as follows: 

HOMA-IR= [fasting glucose (mmol/L) × fasting insulin (µIU/mL) / 22.5]

HOMA β-cell function = [20 × fasting insulin (µIU/mL) / fasting glucose (mmol/L) – 3.5]

Insulin sensitivity was estimated using the Quantitative Insulin Sensitivity Check Index (QUICKI) according to equation QUICKI= 1/ (log insulin (μIU/mL) + log glucose (mg/dL)) (19). Low QUICKI indicates low insulin sensitivity, while high QUICKI indicates high insulin sensitivity.

### 3.5. Statistical Analysis

All continuous data were expressed as Mean ± SD. Data analysis was performed using SPSS software for windows version 14. The Kolmogorov–Smirnov test was used to determine the normality of the distribution, and variables were found to be normally distributed. Group means were compared using independent-samples t-test. Bivariate correlation and linear regression analyses were performed for determining the relationship between serum leptin and other variables and Pearson Correlation Coefficient was obtained. Data were also analyzed by linear regression using leptin as the dependent variable. Independent variables including BMI, body fat percentage, insulin, hip and waist circumferences, fasting plasma glucose, HOMA β-cell function, and HOMA-IR were forced into the model. For all assessments a value of P < 0.05 was statistically accepted as significant.

## 4. Results

Anthropometric, metabolic, and clinical characteristics of the two groups are shown in Table 1. The mean age and BMI for diabetic group were (42.09 ± 6.07 years and 24.35 ± .81 kg/m^2^, respectively) and those for non-diabetic group were (40.07 ± 7.29 years and 24.96 ± .89 kg/m^2^, respectively) (P > 0.05). On average, women were younger, and had higher BMI and better lipid profiles compared to men in both groups, Also, women showed higher insulin concentrations, HOMA-IR, and HOMA-β cell function compared to men in both groups ( Table 2 ).

**Table 1:** Anthropometric Indices and Clinical Characteristic in Non-Diabetic and Diabetic Groups a

	Non-Diabetic	Diabetic	P value b
**Age, y**	40.0 ± 7.2	42.0 ± 3.9	0.126
**Weight, kg**	85.8 ± 10.0	84.1 ± 6.2	0.387
**Height, cm**	1.7 ± 0.0	1.7 ± 0.0	0.619
**BMI, kg/m**^2^	24.9 ± 0.8	24.3 ± 0.8	0.283
**Waist, cm **	108.2 ± 12.2	104.3 ± 10.1	0.125
**Hip , cm**	116.5 ± 6.6	113.7 ± 9.9	0.145
**Waist/Hip**	0.90 ± 0.09	0.9 ± 0.1	0.490
**FPG, mg/dL**	94.7 ± 13.4	158.8 ± 69.3	0.000
**Total cholesterol, mg/dL**	203.6 ± 53.3	180.4 ± 44.9	0.039
**Triglyceride, mg/dL**	167.6 ± 36.4	188.6 ± 52.7	0.043
**LDL cholesterol, mg/dL**	132.4 ± 50.1	109.4 ± 43.1	0.031
**HDL Cholesterol, mg/dL**	39.0 ± 7.6	33.9 ± 5.0	0.001
**HbA1c, %**	4.9 ± 0.4	7.5 ± 2.1	0.000
**Fasting Leptin, ng/mL**	32.1 ± 14.3	19.3 ± 11.4	0.000
Men	23.1 ± 11.0	13.5 ± 8.9	0.005
Women	39.8 ± 12.3	24.8 ± 10.9	0.000
**Fasting Insulin, µIU/mL**	18.2 ± 7.4	21.7 ± 10.0	0.084
**HOMA-IR**	4.3 ± 2.1	8.5 ± 5.9	0.000
**QUICKI**	0.3 ± 0.03	0.3 ± 0.03	0.043
**HOMA-β %**	4.2 ± 2.3	2.8 ± 1.8	0.007
**Body Fat, %**	40.9 ± 7.7	37.4 ± 7.5	0.43

^a^Data are means ± SD

^b^P ≤ 0.05 is considered significant

Abbreviations: BMI, Body mass index; FPG, Fasting plasma glucose; LDL:, Low- density lipoprotein; HDL, High-density lipoprotein; HbA1c, Hemoglobin A1c; HOMA-IR:, Homeostasis Model Assessment for insulin resistance; QUICKI, Quantitative insulin sensitivity check index; HOMA- β, Homeostasis Model Assessment for β-cell function

**Table 2:** Anthropometric Characteristics and Metabolic Parameters in Men and Women of Diabetic and Non-Diabetic Groups a.

	Diabetic		Non-Diabetic	
	Men (n=20)	Women (n=21)	Men (n=18)	Women (n=21)
**Age, y**	42.6 ± 3.7 c	41.5 ± 4.2	40.1 ± 6.3 c	40.0 ± 8.1
**Weight, kg**	75.8 ± 4.4 c	68.0 ± 5.1	76.5 ±6.6 c	70.2 ± 5.7
**Height, cm**	157.8 ± 3.7 c	167.5 ± 5.2	175.6 ± 4.4 c	166.6 ± 5.6
**BMI, kg/m**^2^	24.1 ±0.7 c	24.5 ±0.8	24.7 ±0.9 c	25.1 ± 0.8
**Waist, cm**	107.2 ± 11.7 c	101.5 ± 7.7	111.7 ± 13.8 b	105.1 ± 10.0
**Hip, cm**	110.4 ± 6.6 c	117.0 ± 11.5	114.8 ± 5.1 c	118.0 ± 7.6
**Waist/Hip**	0.9 ± 0.1 b	0.87 ± 0.11	0.94 ± 0.1 b	0.86 ± 0.1
**FPG, mg/dL**	137.6 ± 43.3 c	179.0 ± 83.3	94.0 ± 13.8 b	95.3 ± 13.4
**Total cholesterol, mg/dL**	183.2 ± 41.6 c	177.6 ± 49.0	204.3 ± 58.1 c	203.0 ± 50.3
**Triglyceride, mg/dL**	193.4 ± 49.0 c	184.0 ± 56.9	175.9 ± 32.7 c	160.5 ± 38.7
**LDL-cholesterol, mg/dL**	111.7 ± 39.4 c	107.1 ± 47.6	133.7 ± 53.3 c	131.2 ± 48.4
**HDL- Cholesterol, mg/dL**	32.7 ± 5.8 c	35.0 ± 4.0	38.1 ± 8.5 c	39.8± 6.9
**HbA1c,%**	8.1 ± 2.5 c	7.0 ± 1.7	4.9 ± 0.4 c	4.9 ± 0.3
**Fasting Leptin, ng/mL**	13.5 ± 8.9 b	24.8 ± 10.9	23.1 ± 11.0 b	39.8 ± 12.3
**Fasting Insulin, µIU/mL**	19.5 ± 8.6 c	23.7 ± 10.1	17.4 ± 7.1 c	18.9 ± 7.7
**HOMA-IR**	6.6 ± 3.8 c	10.3± 7.0	4.3 ± 2.2 c	4.5 ± 2.2
**QUICKI**	0.30 ± 0.04 c	0.28 ± 0.0	0.31 ± 0.02 c	0.3 ± 0.04
**HOMA-β, %**	91.5 ± 62.6 c	120.1 ± 91.9	198.5 ± 92.5 c	267.3 ± 119.1
**Body Fat, %**	31.9 ± 6.9 b	42.5 ± 3.5	35.6 ±7.8 b	45.5± 3.8

^a^Independent-samples t-test was used to compare each variable between men and women in each group.

^b^The mean difference between men and women was significant when compared in each group (P < 0.05).

^c^The mean difference between men and women was not significant when compared in each group (P >0.05).

Abbreviations:BMI, Body mass index; FPG, Fasting plasma glucose; LDL, Low- density lipoprotein; HDL, High-density lipoprotein; HbA1c, Hemoglobin A1c; HOMA-IR, Homeostasis Model Assessment for insulin resistance; QUICKI, Quantitative insulin sensitivity check index; HOMA- β, Homeostasis Model Assessment for β-cell function

Comparing two groups regarding age, BMI, height, weight, waist-to-height rate (WHR), waist and hip circumferences, and insulin, no differences were observed. However, the mean concentration of total cholesterol, LDL and HDL- cholesterol, leptin, QUICKI, and HOMA-β cell function in non-diabetic control group were significantly higher than those in diabetic group (P < 0.05) ( Table 1 ). In all of the study subjects mean basal leptin concentration was 25.58 ± 14.38 ng/mL (range 3.10-54.30 ng/mL). Mean ± SD serum leptin concentration was significantly lower in subjects with T2DM compared to non-diabetic control group (19.32 ± 11.43 vs. 32.16 ±14.34 ng/mL). This was observed in both diabetic men (P = 0.005) and women (P = 0.000) compared to non-diabetic control individuals (Table 1).

Serum leptin levels of non-diabetic control subjects ranged from 5.10 to 54.30 ng/mL. Men demonstrated mean serum leptin level of 23.16 ± 11.02 ng/mL ( Table 2 ). On average , women exhibited increasing leptin levels about twice compared to men, showing values of 39.88±12.31 ng/mL (P < 0.0001)even after the levels were normalized based on BMI. When the results were expressed in terms of leptin/BF ratio, this difference was less prominent (1.33- times of men’s for leptin/BF compared to 1.72-times of men’s for leptin/BMI). Despite of no difference in mean BMI (all women, 24.66 ±.96 kg/m^2^; all men, 24.64 ± 0.83 kg/m^2^), mean plasma leptin concentrations of women were almost double compared to those of men (all women, 32.35 ± 13.8 ng/mL; all men, 18.10 ± 10.99 ng/mL). Also, in sex adjusted analysis, the mean concentration of serum leptin in diabetic (24.81±10.95 vs. 13.55 ± 8.97 ng/mL) and non-diabetic women (39.88 ± 12.31 vs. 23.16 ± 11.02 ng/mL) were significantly higher than those of men in both groups (P < 0.001) ( Table 2).

We have calculated body fat content (%) based on the method of Lean et al. using the formula involving age and waist circumference. The body fat percentage in non-diabetic and diabetic subjects were 40.94 ± 7.74% and 37.41 ± 7.59%, respectively (Table 1). Body fat percentage of women was about 1.5-times higher than that of men in diabetic and non-diabetic groups (Table 2). As expected, HOMA calculations showed that type 2 diabetic subjects had a significant increase in insulin resistance (P = 0.001, age and BMI adjusted data) and impaired β cell function (P = 0.007) (Table 1). Furthermore, HOMA-β cell function was lower in poorly controlled diabetic (70.10 ± 46.41) than in well-controlled (129.28 ± 87.50) subgroups (P = 0.017). It was suggested that this subgroup exhibited more severe deterioration of their β cell function compared to the other subgroup. HOMA-β cell function was also lower in men when compared to women in diabetic group (91.58 ± 62.65 vs. 120.10 ± 87.91.46).

**Figure 1:**
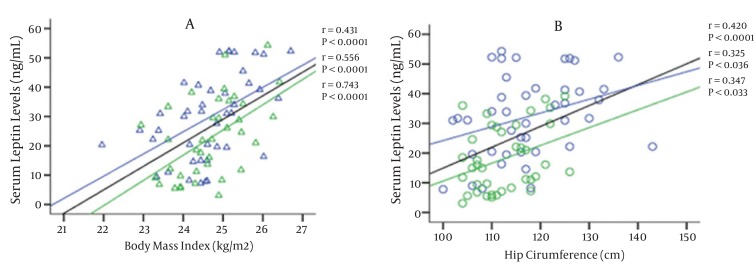
Association Between Serum Leptin Levels and Body Mass Index and Hip Circumference A. Association between serum leptin levels and body mass index. Fasting serum leptin levels of women (blue triangle) upper line and men (green triangle) lower line and total subjects middle line were plotted against body mass index. The "r" values represent correlation coefficient in linear regression analysis. B. Association between serum leptin levels and hip circumference. Fasting serum leptin levels of women (blue triangle) upper line and men (green triangle) lower line were plotted against hip circumference. The ‘r’ value represents correlation coefficient in linear regression analysis.

Figure 1A represents the relationship between serum leptin levels and body mass index in all study subjects. Serum leptin was positively correlated with BMI (r = 0.556, P < 0.0001) in all subjects including men (r = 0.743, P < 0.0001) and women (r = 0.431, P < 0.0001). Serum leptin level was significantly related to waist to hip ratio(r = -0.352, P =0.001). On the other hand, serum leptin showed a correlation with hip circumference in women (r = 0.325, P =0.036) and men (r = 0.347, P = 0.033) as seen in Figure 1B. Serum leptin was linearly related to calculated body fat percentage in all subjects (r = 0.525, P < 0.001) and in men (r = 0.402, P < 0.01).

In the total 80 subjects, bivariate correlation analysis yielded highest correlation of serum leptin to BMI (r = 0.658, P < 0.0001), β cell function (r = 0.577, r < 0. 0001), and calculated body fat percentage (r = 0.431, p < 0.0001) followed by hip (r = 0.420, P < 0.0001), and lastly to insulin (r = 0.226, P < 0.05). Waist to hip ratio, an indicator of abdominal obesity did exhibit negative significant correlation with leptin in the total study subjects(r = -0.352, P = 0.001). In the diabetic subjects only HOMA-IR (r = 0.422, P = 0.006) was related to leptin in addition to BMI (r = 0.597, P < 0.0001).

On the other hand, in non-diabetic subjects only body fat percentage (r = 0.466, P = 0.003) was related to leptin in addition to BMI (r = 0.661, P < 0.0001). Therefore, the main differences due to diabetic status were a significant relationship between leptin, and HOMA-IR. Thus, we found that only in subjects with type 2 diabetes, serum leptin levels depend on HOMA-IR calculated using insulin and glucose concentrations. On the other hand, serum leptin levels depend on body fat percentage as an indicator of visceral obesity only in non-diabetic subjects. Our results suggest that serum leptin levels in non-diabetics might be influenced differently from that in diabetics. In non-diabetics, leptin levels were partly contributed to increased body fat percentage calculated by waist circumference, an indicator of abdominal obesity.

Table 3 represents the results on linear regression analysis carried out using serum leptin as the dependent variable. In all subjects, serum leptin levels were contributed to BMI, βcell function, and gender. Also these variables were the predictors of leptin in normal subjects. On the other hand, in diabetic subjects, serum leptin levels were also predicted by BMI, insulin, and gender. Gender specific regression analysis revealed that in women changes in leptin levels were explained by βcell function and BMI while in men only by BMI. Our results suggest that serum leptin levels in women are influenced differently from those of men. Men had more severe deterioration of their β cell function compared to women. On the other hand, in women insulin levels were partly contributed to increased β cell function which in turn influences leptin levels. This was a novel finding in our study.

**Table 3:** Linear Regression Analysis Using Leptin as Dependent Variable a

	B	S.E (E)	P
**Total (n = 80)**			
BMI	4.928	0.778	0.0001
β cell function	4.217	0.008	0.0001
Gender	-8.621	2.001	0.0001
**Non-diabetic**			
BMI	7.354	1.269	0.0001
Gender	-10.502	2.717	0.0001
β cell function	3.514	0.01	0.002
**Diabetic**			
BMI	3.738	0.901	0.0001
Insulin	0.341	0.128	0.011
Gender	-6.741	2.626	0.014
**Men (n = 38)**			
BMI	5.738	0.860	0.0001
**Women (n = 42)**			
β cell function	5.014	0.011	0.0001
BMI	4.499	1.347	0.002

^a^Independent variables included were: BMI, %body fat, FPG, insulin, HOMA-IR, HOMA-βcell function, Waist, hip, and WHR.

Abbreviations: BMI, Body mass index; FPG, Fasting plasma glucose; HOMA-IR, Homeostasis Model Assessment for insulin resistance; HOMA- β, Homeostasis Model Assessment for β-cell function; WHR, waist to hip ratio

## 5. Discussion

The present study evaluates serum leptin levels in non-obese subjects with type 2 diabetes. We found that leptin levels correlated with BMI and body fat percentage in both diabetic and non-diabetic subjects, and were higher in women than in men which confirm previous studies in other populations (20, 21). The main finding of this study was that non-obese subjects with type 2 diabetes had lower serum leptin concentrations compared to non-diabetic controls, as was previously observed in moderately obese diabetic subjects (7-9). The serum leptin levels were not reduced due to any significant difference in BMI and age because two groups were adjusted for these variables. It is speculated that low serum leptin levels in diabetic patients in our study may be the consequence of relatively defective function of pancreas; which was seen in lower β-cell function determined by homeostatic model assessment for β- cell function as well as highly insulin resistance evident by HOMA-IR values, both of them were consistent with the established features of diabetes (22). Therefore, lower leptin levels could be partly attributed to these metabolic abnormalities.

Reports regarding the role of leptin in diabetes are inconsistent; some studies have reported increased (17) or decreased (23, 24) or unchanged (12) serum leptin levels in diabetic patients. Serum leptin levels are affected by gender, BMI, adiposity, insulin levels, insulin sensitivity, and treatment regimen. Therefore, differences in these variables among different studies may explain conflicting results. According to Wauters et al. (25) stating that adiposity and gender are the main determinants of leptin levels in normal and diabetic patients, therefore, part of the controversy among previous reports could be attributed to the differences in adiposity or gender of the patients. Many investigators have described leptin alterations only in obese or overweight patients (4, 7, 11, 24). Few workers have studied only on men (11) or on women (26). Our results are somewhat similar to two other reports, one in non-obese Indian subjects (23), and another in obese Sudanese patients (25).

Although there was no certain reason for increase or decrease of serum leptin levels in type 2 diabetes compared to non-diabetics, a possible explanation for reducing leptin levels in diabetic subjects is a difference in the fat distribution between 2 groups which was not determined in this study. It was shown that subcutaneous fat produces more leptin compared to omental fat (27) and that diabetics own more visceral and less subcutaneous fat; these considerations were not determined in current study. This would then confirm a previous report of lower leptin levels in diabetics of Caucasian origin due to altered fat distribution (28). Another possibility, however, is a relative insulin deficiency in diabetic subjects, because insulin is an important stimulator of leptin production (5, 25, 29). Thus, as calculated by HOMA model, the diabetic subjects had a marked insulin resistance and impaired insulin secretion suggesting a relative insulin deficiency.

The strong relation between body mass index and plasma leptin was previously reported by many investigators for non-diabetic (1), insulin resistant (30), or type 2-diabetic subjects (4, 31). In our study, also, leptin had a strong correlation with BMI in non-diabetic and diabetic groups.

As a result, we found a positive and significant correlation between leptin and hip circumference in diabetic and non-diabetic groups. Inversely, there was a negative and significant correlation between serum leptin concentration and waist to hip ratio in both groups. These finding are consistent with results reported by other investigators (26, 32).

In this study, the leptin levels in women were significantly higher than those in men in both groups, which was a similar finding to those in previous reports on non-diabetic and diabetic subjects. Investigators reported serum leptin level was higher in women than in men (32-35) and this is probably owing to the adipose tissue in women being more compared to men, the existence of negative correlation between leptin and testosterone levels (33, 35), and the stimulation of leptin mRNA production by 17β-estradiol, which is one of the women’s sexual hormones (36).

The relationship between insulin and leptin has been studied extensively and reviewed previously (37). In Caucasian patients with type 2 diabetes who were also overweight and middle-aged, leptin levels were significantly associated with insulin secretion and insulin resistance (16), and with insulin secretion in patients undergoing oral drug therapy (38). Increased leptin levels following insulin treatment in rodents (39) and in diabetic patients (40) suggest the insulin deficiency as the cause of altered leptin levels in diabetes. Similar to our data, leptin levels in lean diabetic women from Bangladesh were reported to be related to fasting insulin in diabetic women (41). Results from obese Sudanese patients suggest that HOMA-IR and -β cell function are related to leptin in diabetes (13).

The only limitation of our study was the sample size. It is suggested that further studies involving large number of patients of both sexes with direct quantification of body fat content are needed to understand the role of leptin in details in diabetic patients. Briefly, our results suggest that serum leptin levels in women are influenced differently from those of men. Men had a more severe deterioration of their β cell function than that of women. On the other hand, in women insulin levels are partly contributed to increased β cell function which in turn influences leptin levels. This is a novel finding in our study. Moreover, we speculated that the reduction serum leptin levels in type 2 diabetes and even lower levels in subjects with poorly controlled diabetes was likely due to male gender, insulin deficiency, and defect of β-cell function. Furthermore, sample size studies, which should investigate the molecular mechanisms, are needed to make clear the issue for reduced serum leptin levels, whether it is a reason or an outcome.
